# The effects of note-taking methods on lasting learning: the role of motivation and cognitive load

**DOI:** 10.3389/fpsyg.2025.1697151

**Published:** 2026-01-21

**Authors:** Mesut Yıldırım

**Affiliations:** Department of Educational Sciences, Harran University, Şanlıurfa, Türkiye

**Keywords:** cognitive load, experimental study, lasting learning, learning motivation, note-taking methods

## Abstract

**Introduction:**

This randomized controlled experimental study examined the effects of four note-taking methods (Cornell, Parallel, Digital, and Sentence) on lasting learning (retention) in primary-level teacher candidates, while also exploring the roles of learning motivation and cognitive load.

**Methods:**

A total of 134 participants were randomly assigned to three experimental groups (Cornell, Parallel, Digital) and one control group (Sentence). Following a one-session orientation, the intervention spanned 5 weeks (2 h/week). Measures included a researcher-developed Academic Achievement Test (pre-, post-, retention), a validated Learning Motivation Scale (pre-, post-), and a single-item cognitive load rating collected after each lesson. Analyses used mixed ANCOVA with Bonferroni-corrected post-hoc tests and regression analyses.

**Results:**

Mixed ANCOVA revealed significant main effects of time and group, whereas the time × group interaction was not significant. Post-hoc tests showed no significant pairwise differences at post-test; at retention, only the Cornell group scored significantly higher than the Sentence group. Motivation increased significantly in the Cornell and Parallel groups, while Digital and Sentence showed no significant pre–post change. Cognitive load differed by method, with Digital reporting significantly lower load than Parallel and Sentence. Regression analyses indicated that motivation was significantly associated with retention, whereas cognitive load was not.

**Conclusions:**

Overall, retention differences were limited and observed only for the Cornell–Sentence comparison. Learning motivation showed the most consistent association with retention scores, whereas cognitive load showed no significant association with retention in the regression analyses.

## 1 Introduction

In contemporary higher education, one of the central instructional challenges is not only to promote short-term academic performance but to foster *lasting learning*—knowledge that remains accessible and usable weeks or months after initial instruction. Research in the learning sciences suggests that although learners often demonstrate immediate gains, much of this knowledge can diminish over time—particularly under massed or performance-oriented study conditions—yet the extent of this decline varies across tasks, contexts, and learner characteristics (Richter et al., 2022; Russ et al., 2025a; Kim et al., 2025). For pre-service teachers, whose professional competence depends on the durable mastery of pedagogical principles, subject-matter knowledge, and instructional strategies, understanding how to support learning that endures beyond the short term is of both theoretical and practical importance.

Over the past two decades, cognitive and educational psychology have identified several principles—such as spacing, retrieval practice, elaboration, and successive relearning—that reliably strengthen long-term retention (Cepeda et al., 2006; Karpicke and Blunt, 2011; Roediger and Karpicke, 2006). Although robust evidence demonstrates that spaced retrieval enhances retention across age groups, domains, and authentic learning environments (Dunlosky et al., 2013; Karpicke and Blunt, 2011), these strategies remain underused in everyday study routines. Many learners rely instead on rereading or last-minute cramming, practices that support short-term performance but seldom lead to lasting learning (Roediger and Karpicke, 2006). This persistent gap between research-based strategies and typical study behaviors underscores the need to explore *instructional routines* that naturally embed effective learning principles into regular teaching. Note-taking is one such ubiquitous routine.

Classic and recent work suggests that note-taking can foster deeper processing, integration, and organization of information, particularly when students transform and structure incoming content rather than transcribing it verbatim (Mueller and Oppenheimer, 2014; Bui et al., 2013). From the perspective of the ICAP framework (Interactive, Constructive, Active, Passive), note-taking can shift students from passive reception toward more active or constructive engagement, which reliably predicts stronger learning outcomes (Chi and Wylie, 2014). However, note-taking is inherently heterogeneous: structured formats such as the Cornell system, two-column/parallel notes, digital environments, and linear sentence-based notes differ in the extent to which they support generative processing, self-questioning, and efficient review.

Empirical findings on note-taking methods remain mixed. One meta-analysis reported little difference between longhand and digital notes when distractions were controlled (Voyer et al., 2022), whereas another found that handwritten notes produced higher conceptual performance despite typed notes yielding more volume (Flanigan et al., 2024). These inconsistencies suggest that medium alone is insufficient to explain learning outcomes and that motivational and cognitive factors may account for variability across note-taking contexts. Cognitive Load Theory (CLT) emphasizes that instructional formats differ in the degree to which they impose intrinsic, extraneous, and germane cognitive load (Sweller et al., 2011). Structured note-taking formats may reduce extraneous load by clarifying organization, but they may also introduce “desirable difficulties” that increase effort in ways that ultimately benefit long-term retention (Bjork and Bjork, 2011; Sweller et al., 1998).

Motivation also shapes cognitive effort, strategy use, and engagement. Instructional environments that support autonomy, competence, and meaning can sustain the effortful processing required for deeper learning (Ryan and Deci, 2020; Schunk and DiBenedetto, 2020). Thus, understanding how learners’ motivation and perceived cognitive load interact with note-taking methods is essential for explaining why certain methods may support immediate performance while others foster more durable retention.

Although note-taking, motivation, and cognitive load have each been studied extensively, there is a notable lack of research examining how these three variables jointly influence immediate and lasting learning within the same experimental design, particularly in naturalistic university settings. Most studies rely on short, single-session laboratory tasks that limit insight into how note-taking practices function *over time* during authentic coursework. Moreover, studies comparing note-taking methods seldom include delayed retention assessments, leaving unanswered which strategies genuinely promote learning that endures beyond initial instruction. Consequently, there remains a clear need for ecologically valid, longitudinal research that integrates note-taking method, motivation, and cognitive load to explain both short-term performance and the durability of learning. The present study addresses this gap and, in doing so, builds upon the theoretical foundations of lasting learning outlined in the following section.

### 1.1 Lasting learning

Lasting learning is defined not merely as the short-term acquisition of knowledge, but as the ability to retain information in meaningful schemas within long-term memory, allowing it to be remembered over time and transferred to novel situations. In this context, “lasting” learning encompasses long-term retention strengthened by effective recall processes and flexible application of knowledge (Karpicke and Blunt, 2011; Roediger and Karpicke, 2006).

The literature indicates that lasting learning is particularly supported by four key components:

1. Retrieval practice enhances long-term retention by requiring learners to actively recall information rather than passively reviewing it (Karpicke and Blunt, 2011; Roediger and Karpicke, 2006);
2. Spaced or distributed practice slows the forgetting curve and significantly increases durability by spreading study sessions over time (Cepeda et al., 2006);
3. Elaboration and organization (e.g., conceptual structuring, relational encoding) create more permanent memory traces by linking new information to existing schemas (Dunlosky et al., 2013);
4. Cognitive load management entails designing instruction in a way that considers the limitations of working memory, reducing extraneous load and promoting processing that supports schema construction (Sweller et al., 1998).

Although a substantial body of research shows that lasting learning is often supported by engaging in active recall, distributing learning over time, organizing information meaningfully, and managing cognitive load effectively, empirical findings indicate that these effects do not consistently emerge across instructional contexts. Recent work demonstrates that even well-established principles such as spaced or distributed practice can yield attenuated or nonsignificant benefits depending on task characteristics, the nature of instructional materials, and learner attributes. For example, Krauspe et al. (2025) found that spacing failed to reliably enhance long-term retention when learning from worked examples, and Russ et al. (2025b) reported that distributed instruction benefited only students with higher academic self-concept or work ethic, producing no general advantage for the broader sample. Moreover, Higham et al. (2023) showed that under certain conditions restudy outperformed spaced retrieval practice, casting doubt on the universality of retrieval-based advantages. Collectively, these findings suggest that the mechanisms underlying lasting learning operate in a more conditional and context-dependent manner than often assumed. Because note-taking methods can shape how learners encode, structure, and later retrieve information, understanding their impact requires acknowledging both the robust and the variable patterns observed in the literature. This more nuanced perspective provides the theoretical basis for examining how note-taking practices may differentially influence the cognitive and motivational processes associated with lasting learning.

### 1.2 Learning motivation

There are numerous factors that influence lasting learning, and one of these is motivation during the learning process. Learning motivation is defined as “the internal processes that initiate and sustain goal-directed activities,” and it manifests in outcomes such as choice, effort, persistence, and self-regulation (Schunk and DiBenedetto, 2020). According to Self-Determination Theory (SDT), motivation exists along a continuum in terms of quality: at the most autonomous end lies intrinsic motivation; adjacent to it is identified regulation, which is based on personal value attribution; further along the controlled end are introjected regulation (internalized pressure) and external regulation; and at the farthest end lies amotivation (Ryan and Deci, 2020).

Motivation enhances academic success by supporting the use of cognitive strategies, deep processing, task engagement, and persistence in learning. A meta-analysis demonstrated that autonomous motivation (intrinsic + identified) is consistently positively associated with achievement, engagement, and well-being, while controlled motivation (introjected + external) is linked to weaker or even negative outcomes (Howard et al., 2021). Moreover, motivation interventions implemented in school settings have shown varying degrees of impact on authentic outcomes such as grades and standardized test scores (Lazowski and Hulleman, 2016).

The literature on lasting learning shows that motivational states strengthen both the encoding and consolidation of learning processes, which in turn supports lasting learning, particularly that associated with long-term memory. In this context, Gruber et al. (2014) demonstrated that intrinsic motivation can enhance the lasting learning of information that is not immediately relevant to a task. Similarly, Duan et al. (2020) found that the combination of intrinsic and extrinsic motivation positively influences the durability of learning, especially in relation to episodic memory. Taken together, these findings suggest that motivation not only affects the level of learning achieved but also the extent to which that learning is retained over time (Bureau et al., 2022; Duan et al., 2020; Endres and Eitel, 2024; Gruber et al., 2014; Howard et al., 2021).

### 1.3 Cognitive load

Another variable that can affect motivation and, consequently, be targeted in interventions for lasting learning is cognitive load during the learning process. Cognitive load refers to the amount of mental effort placed on an individual’s cognitive resources – particularly working memory – during learning. According to Cognitive Load Theory (CLT), working memory has limited capacity, and when the cognitive demands of a task exceed this capacity, learning can be hindered (Zu et al., 2021).

Cognitive load is typically divided into three components. Intrinsic load arises from the inherent complexity of the learning material, reflecting the difficulty level that learners must manage during comprehension. Extraneous load is imposed unnecessarily by ineffective instructional design or by presenting information in a way that does not facilitate understanding. In contrast, germane load refers to the mental effort devoted to learning processes that promote schema construction and enhance meaningful learning.

Effective instructional design aims to manage intrinsic load at an appropriate level, minimize extraneous cognitive load, and maximize germane load that facilitates learning. For example, breaking complex topics into smaller chunks or providing visual and auditory supports can reduce the burden on working memory and thereby enhance learning.

Cognitive Load Theory posits that learners can process information more effectively and transfer it to long-term memory when they are not overwhelmed by excessive cognitive load (Sweller et al., 2011). Indeed, “cognitive load can also be defined as the mental effort experienced in working memory while performing a particular task” (Zu et al., 2021). Maintaining this load at an optimal level helps learners focus on the essential content and engage in deep processing.

### 1.4 Note-taking methods

Note-taking is an indispensable component of the learning process, and an appropriate note-taking method can significantly enhance both information processing and recall. Various note-taking methods have been developed in educational literature. This section conceptually describes four widely used note-taking methods that are also examined within the scope of this study. These include the Cornell note-taking method, which organizes information into structured sections to promote review and summarization; the parallel note-taking method, which allows students to record main ideas and details simultaneously in corresponding columns; the digital note-taking method, which employs technological tools and devices to enhance accessibility and organization; and the sentence method, which involves writing down information in full sentences to capture detailed content and logical flow. These methods were selected because they are frequently used by university students, have been examined in prior literature, and differ theoretically in how they may influence motivation, perceived cognitive load, and both immediate and lasting learning. These four methods differ meaningfully in how they shape learners’ cognitive engagement, the organization of content, and the ease of later review. As such, they provide an appropriate basis for examining how note-taking practices interact with motivation and cognitive load within an extended instructional context.

The core characteristics and effects of each method on the learning process are conceptually explained below.

#### 1.4.1 Cornell note-taking method

The Cornell note-taking method (Cornell note system) was developed in the 1940s by Walter Pauk at Cornell University, aiming to help students take class notes in a more organized and meaningful manner.

**Figure 1:**
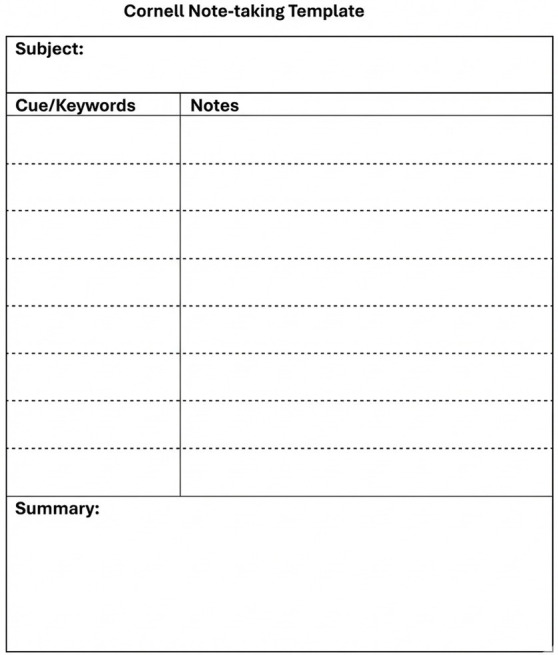
Cornell method template.

In this method, the student divides the note page into two columns: detailed class notes are written on the right side, while key concepts, questions, or cues related to those notes are recorded on the left side (Pauk, 2000). At the end of the lecture or afterwards, a brief summary is added at the bottom of the page (Figure 1).

The distinctive feature of the Cornell method is that it facilitates the student’s ability to review and self-question the notes afterwards. Indeed, this “split-page” technique reinforces learning by encouraging the student to ask and answer questions independently (King, 1991; Nist and Holschuh, 2000). For example, a student might write a question related to the lesson in the left column and then search for the answer in the notes on the right column, effectively testing their own understanding. This process leads to much more effective learning compared to merely reviewing notes passively; students who generate and answer their own questions demonstrate superior comprehension and recall compared to those who simply read the material (King, 1991; Nist and Holschuh, 2000).

Another advantage of the Cornell method is that it allows for quick scanning of key points during review. Thanks to the keywords and questions in the left column, the student can rapidly recall the general framework of the topic. Academic research has shown that the Cornell note-taking method has positive effects on students’ exam performance and knowledge retention (Keleş and Çepni, 2006; Jacobs, 2008).

#### 1.4.2 Parallel note-taking method

The parallel note-taking method, first introduced into the literature by Pardini et al. (2005) in their article describing the simultaneous use of dual-column note-taking with “webnotes,” is based on the principle that while the student follows the webnote or presentation handout used in class in one column, they add their own explanations, examples, questions, and mnemonic cues in the opposite column. The aim is to balance the learner’s divided attention between listening, processing, and writing, thereby promoting active engagement and the production of meaningful notes.

**Figure 2:**
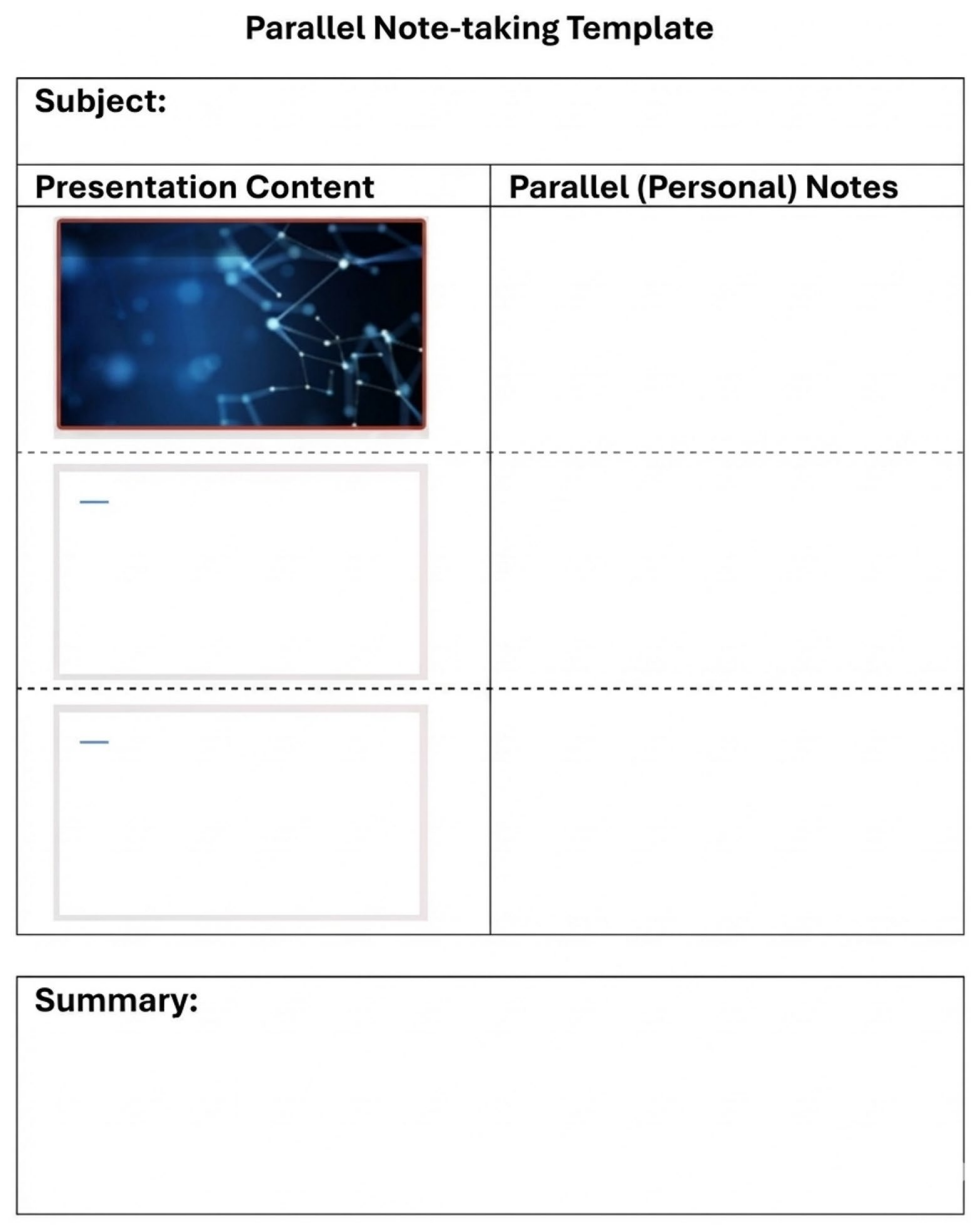
Parallel method template.

The first systematic definition of the method was presented in a study that taught students to take “two-column/side-by-side” notes using presentation slides. Students participating in the implementation found the method effective in terms of engaging with the material and adding elaborations (Pardini et al., 2005). In this method, the student places printed or digital presentation notes—provided by the instructor—on the left side during the lecture, while simultaneously writing their own notes on the right side in alignment with the materials. In other words, while the presentation slides or lecture notes appear on one side of the page, the student adds their own explanations, examples, or reminder notes opposite them or in the page margins (Figure 2).

This approach can also be interpreted as an instructional design aligned with Cognitive Load Theory, aiming to reduce problems such as split attention and unnecessary (extraneous) load. As the core content is provided in a ready-to-follow format, students can allocate their cognitive resources to making connections, generating examples, and engaging in self-regulation (Krapf and Pfefferkorn, 2022).

Recent literature offers consistent findings regarding the effects of parallel note-taking and related structured note formats (e.g., guided notes, double-entry/two-column notes, collaborative notes) on learning. A systematic review conducted with university students reported that, based on studies from 2009 to 2019, the use of guided notes increased note accuracy and, in some cases, retention test scores across various knowledge domains; students also evaluated these materials positively in terms of focus and engagement (Biggers and Luo, 2020). In a study conducted in an undergraduate mathematics course, students preferred guided notes with blanks over no notes or full notes, stating that this format helped maintain attention, facilitated information processing/storage, and enhanced interaction during lectures (Krapf and Pfefferkorn, 2022). Similarly, double-entry note-taking has been shown to activate higher-order cognitive processes by supporting students’ critical reading of texts and connection of multiple sources (Ives et al., 2020). Moreover, a 2024 study demonstrated that the positioning of blanks and pre-filled sections in guided notes influenced student preferences and usability, with well-designed templates improving attention and tracking (Feudel and Panse, 2024).

One of the recent findings that explains the effects of parallel note-taking at the mechanistic level is its impact on cognitive load. In an experimental study comparing three different note-taking styles, students using collaborative/structured notes achieved higher learning outcomes and reported lower perceived cognitive load. The researchers attributed this result to the structured formats’ ability to reduce extraneous load sources such as split attention and redundancy (Shi et al., 2022). Since parallel note-taking presents the instructional material in one column as a “scaffold” and allows the student to generate their own elaborations in the other column, it enhances both the quality of the note product and the immediacy of in-class processing (Krapf and Pfefferkorn, 2022; Pardini et al., 2005).

#### 1.4.3 Digital note-taking method

**Figure 3:**
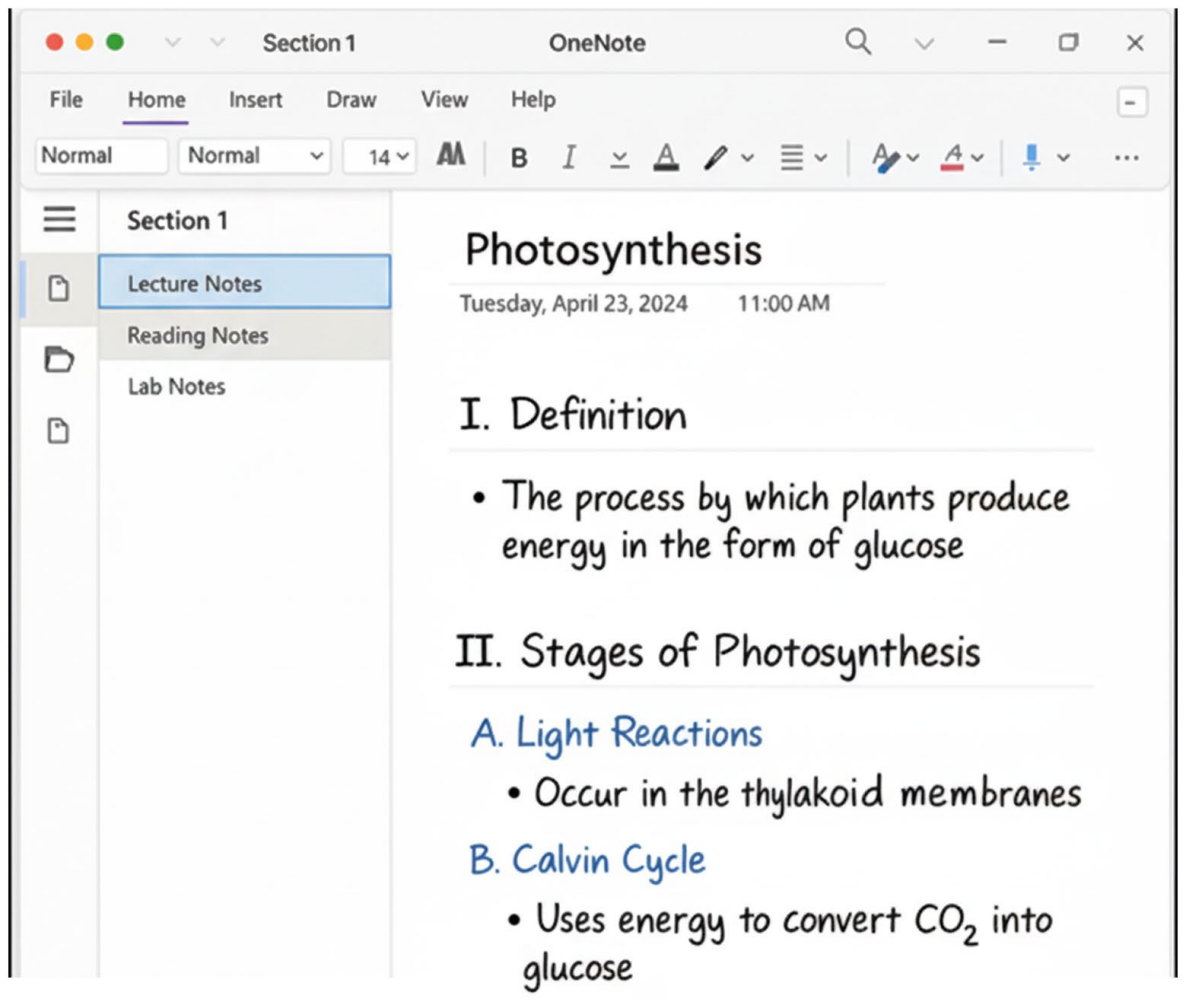
Digital method (OneNote example).

Digital note-taking refers to the process of recording information during lectures or readings using digital tools such as computers, tablets, or smartphones, typically via keyboard or stylus input, followed by the organization and possible sharing of these notes. The development of the digital note-taking method is closely linked to technological advancements. With the emergence of personal computers in the 1960s and the widespread use of word processors from the 1980s onward, students began transferring their written notes into digital formats. The proliferation of software such as Microsoft Word, OneNote, and Evernote throughout the 1990s and 2000s further popularized digital note-taking (Figure 3).

Today, portable devices like iPads and Android tablets, along with applications such as Apple Notes, Google Keep, Notion, and Obsidian, as well as stylus-supported tools (e.g., Apple Pencil, Samsung S Pen), have turned digital note-taking into a widely adopted learning strategy. In the literature, this process is evaluated within the context of the evolution of learning technologies (Bond, 2020; Mueller and Oppenheimer, 2014). This progression illustrates that digital note-taking is no longer viewed solely as a method of recording, but also as a cognitive learning strategy (Bui et al., 2013; Luo et al., 2018).

Three key stages of practice are emphasized:

1. Preparation: Pre-creation of folders/templates (headings, bullet points, two-column layouts, etc.); turning off distracting notifications.
2. Real-time capture: Recording of key concepts during class or reading using hierarchical headings, bullet points, and ideally immediate elaborations (restated in the student’s own words).
3. Consolidation: Post-lesson summary writing, adding keywords/tags, enabling searchability, and engaging in retrieval practices (e.g., brief self-testing).

Digital notes offer advantages such as speed and ease of editing (searching, copying, reordering), the ability to integrate multimedia elements (links, images, screenshots), and opportunities for collaborative work (shared documents). These features particularly enhance accessibility and review in content-heavy contexts (Voyer et al., 2022). However, when taking notes via keyboard, students are often inclined toward verbatim transcription, which can lead to shallow processing and reduced performance on conceptual questions (Mueller and Oppenheimer, 2014). Moreover, digital environments elevate the risk of multitasking and divided attention; laptop use in class can negatively affect both the users’ and nearby students’ learning (Sana et al., 2013). In contrast, using structured templates (e.g., two-column layouts, cue–response formats, guided notes) and disabling distractions can reduce verbatim tendencies and extraneous cognitive load, thereby improving outcomes (Voyer et al., 2022). In sum, digital note-taking contributes most effectively to lasting learning when the prepare–capture–consolidate sequence is followed with discipline and combined with restatement in one’s own words and retrieval practices (Mueller and Oppenheimer, 2014; Voyer et al., 2022).

#### 1.4.4 Sentence note-taking method

**Figure 4:**
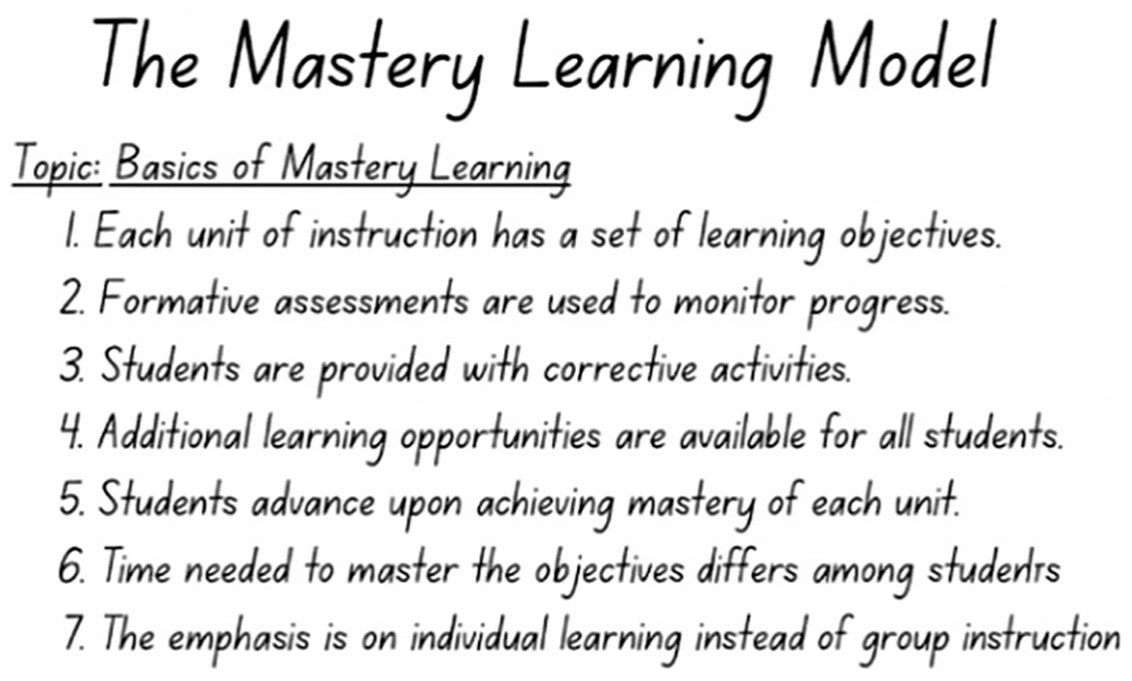
Sentence method example.

The sentence method is one of the most basic and traditional note-taking methods. It is based on the principle of recording each piece of information or idea as a separate sentence written sequentially. The sentence note-taking method is defined as writing each important piece of information heard during a lecture or reading as a short sentence, mostly in chronological order, and postponing the organization into headings and subheadings until the review phase (Figure 4).

It is considered a variant of verbatim-oriented strategies, which have been studied since the early stages of research on study skills and note-taking (Kobayashi, 2006). Since this method does not require any specific format or structure, it allows for rapid note-taking. During fast-paced lectures, the student can attempt to capture as much information as possible without spending time organizing the hierarchical structure of the content. In this regard, it is effective in capturing a large number of ideas (Bui et al., 2013; Luo et al., 2018).

The main limitation of this method, also seen in digital note-taking, is its tendency to promote verbatim transcription (Mueller and Oppenheimer, 2014). Additionally, long strings of unorganized sentences may later increase extraneous cognitive load, potentially making it more difficult to organize and retrieve the content from memory (Kobayashi, 2006). Some studies in the literature have shown that the sentence method is functional in situations requiring speed and breadth; however, it becomes more effective for lasting learning and deep comprehension when supported by post-lecture steps such as summarization, review, and reorganization (Kobayashi, 2006; Mueller and Oppenheimer, 2014; Shi et al., 2022).

### 1.5 The current study

The main purpose of this research is to determine the effects of note-taking methods on lasting learning, while the secondary purpose is to reveal how lasting learning is influenced by learning motivation and cognitive load variables within the scope of note-taking methods. In this regard, the study aims to answer the following research sub-questions:

1. Is there a statistically significant difference between the post-test and retention test academic achievement scores of the experimental and control groups when the pre-test scores are controlled?
2. Is there a significant difference between the pre-test and post-test scores of learning motivation in the experimental and control groups?
3. Is there a significant difference between the experimental and control groups in terms of mean cognitive load?
4. Do motivation and cognitive load significantly predict lasting learning based on the note-taking methods used?

## 2 Research methods

### 2.1 Research model

In this study, an experimental model was employed, specifically a pre-test/post-test control group design with a multi-group mixed experimental design (Table 1).

**Table 1:** Experimental design.

Groups	Assignment	Experimental treatment	Pre-test	Follow-up test	Post-test	Retention test
Experimental 1	R	Cornell NtM	S_1_, S_2_	S_3_	S_1_, S_2_	S_1_
Experimental 2	R	Parallel NtM	S_1_, S_2_	S_3_	S_1_, S_2_	S_1_
Experimental 3	R	Digital NtM	S_1_, S_2_	S_3_	S_1_, S_2_	S_1_
Control group	R	Sentence NtM	S_1_, S_2_	S_3_	S_1_, S_2_	S_1_

R, random assignment; NtM, note-taking method.

S_1_, Academic Achievement Test; S_2_, Learning Motivation Scale; S_3_, Cognitive Load Scale.

A total of three experimental groups and one control group were formed, with participants randomly assigned to groups.

- Between-groups factor: four groups

*Exp. 1 → Cornell; Exp. 2 → Parallel; Exp. 3 → Digital; Control → Sentence*

- Within-groups factor (time): three measurement points

*Pre-test, Post-test, Retention test.*

### 2.2 Study group

In the study, homogeneous sampling, a type of purposeful sampling method, was employed. Homogeneous sampling is based on selecting a subgroup from the population that shares similar characteristics with respect to a particular variable and examining the data obtained from this group in detail (Büyüköztürk et al., 2014; Yıldırım, 2022). In this context, homogeneity was ensured by including students who were all enrolled in the second year of the Faculty of Education. Accordingly, the study group consists of 134 s-year students enrolled at the Faculty of Education, Harran University, in 2024. These students voluntarily agreed to participate in the study, and they were between 18 and 22 years old, representing a relatively homogeneous age group typical of second-year pre-service teachers. Within the scope of the research, the students were assigned to three experimental groups and one control group through random assignment, and the distribution is presented in Table 2.

**Table 2:** Distribution of participants across experimental and control groups.

Groups	*N*	%
Experimental 1: Cornell NtM	32	23.9
Experimental 2: Parallel NtM	33	24.6
Experimental 3: Digital NtM	33	24.6
Control: Sentence NtM	36	26.9
Total	134	100.0

According to the table, it is observed that the number of students in the experimental and control groups is relatively close (ranging between 23.9 and 26.9%). In some quantitative analysis techniques, having no large differences in group sizes is considered desirable. Therefore, care was taken to ensure a balanced distribution.

### 2.3 Study design and intervention

**Figure 5:**
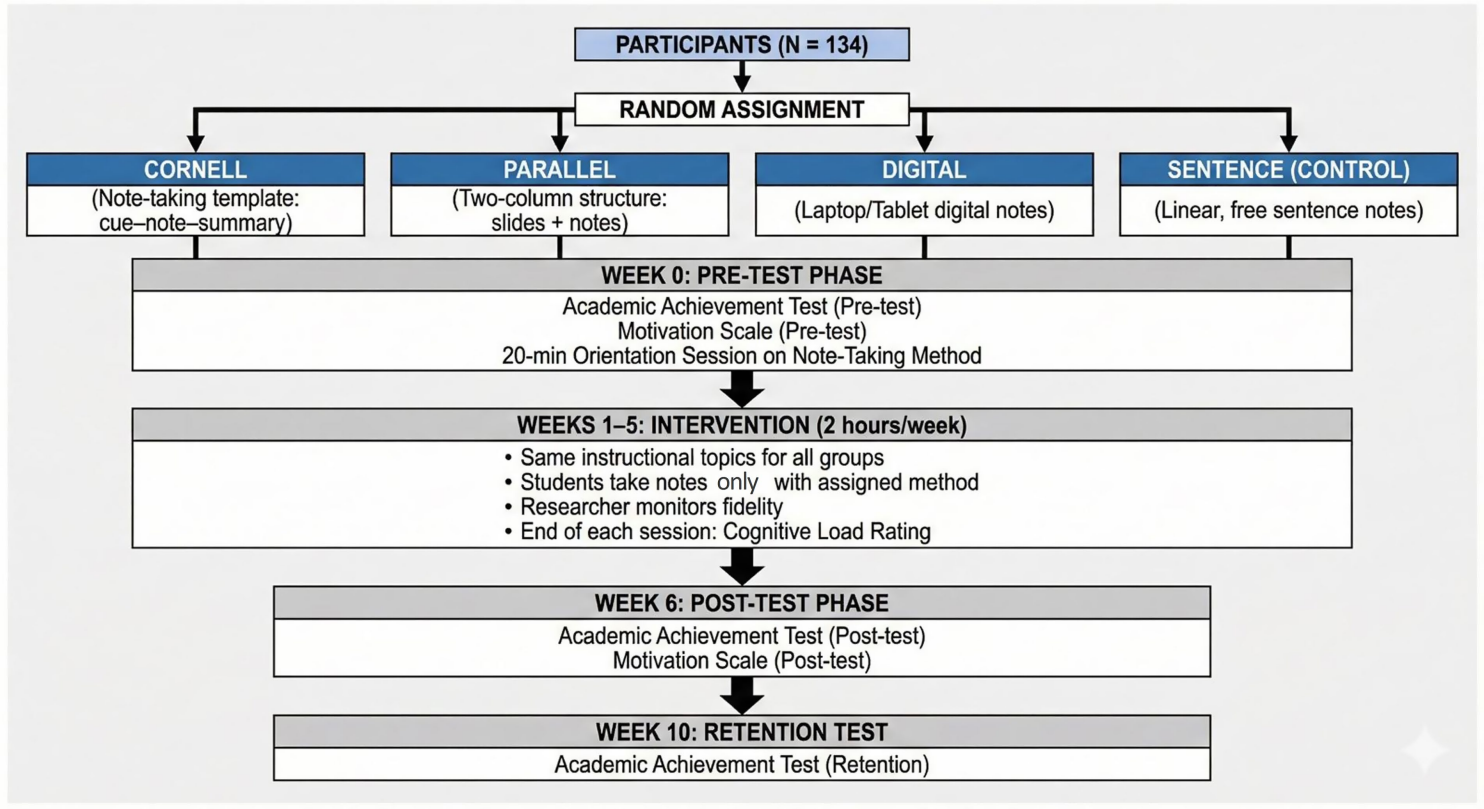
Study procedure timeline.

- The study employed a randomized controlled pre-test–post-test control group design with four groups (Cornell, Parallel, Digital, and Sentence). Participants were randomly assigned to these groups to ensure equivalence at baseline.
- The instructional content delivered during the five-week intervention was drawn from the course “Instructional Principles and Methods,” a core module for second-year pre-service teachers. All groups covered identical weekly topics, including direct instruction, cooperative learning, inquiry-based teaching, assessment strategies, instructional planning, and learner-centered approaches. The same presentation materials, examples, and explanations were used across groups to ensure instructional consistency.
- Prior to the intervention, all groups completed the Academic Achievement Test and the Motivation Scale as pre-tests.
- Before beginning the intervention, each group was provided with a short (approximately 20-min) orientation session introducing the note-taking method assigned to them. The implementation of these methods differed by group:
  - Cornell method: Students used a cue–note–summary template, writing keywords/questions in the left column, detailed explanations in the right column, and completing a short summary at the bottom of the page after each lesson.
  - Parallel (two-column) method: The left column contained presentation slides or instructor-provided outlines, while students recorded their own elaborations, examples, and explanatory notes in the corresponding right column.
  - Digital method: Students used either laptops or tablets to take notes with digital tools (e.g., OneNote). Templates were recommended, but students were allowed to adjust formatting as long as the structure remained consistent.
  - Sentence method (Control): Students took linear, unstructured sentence-based notes without any imposed organization.
  - Throughout the five-week intervention (2 h per week), students were required to take notes exclusively using the method assigned to their group. The researcher monitored adherence to ensure fidelity to the instructional design.
  - At the end of each weekly session, students rated their perceived cognitive load using an online form.
  - One week after the intervention concluded, all groups completed the Academic Achievement Test and the Motivation Scale as post-tests.
  - Four weeks after the post-test, the Academic Achievement Test was administered again as a retention test to evaluate lasting learning (Figure 5).

### 2.4 Data collection

Within the scope of the research, various measurement tools were employed to serve as pre-test, post-test, and retention test, and follow-up test.

#### 2.4.1 Academic Achievement Test

The test was developed by the researcher. A pilot study was conducted with 178 students, and item analyses were carried out using the 27% upper–lower group method. Within this framework, item difficulty, item discrimination, and reliability were established. The final form consisted of 20 multiple-choice questions with five options each. The items were designed to assess knowledge of instructional strategies, methods, and techniques.

Initially, the questions were reviewed for content validity by two experts from the field of curriculum and instruction, who rated each item as appropriate or not appropriate. Expert evaluations were analyzed using Cohen’s Kappa coefficient, and as a result of these analyses, the Content Validity Index (CVI) was determined to be 0.814.

The item analyses related to the achievement test are presented (Table 3).

**Table 3:** Item and test analyses of the Academic Achievement Test.

Item num	*p*_j_	*r*_jx_	*r*_x_	Item num	*p*_j_	*r*_jx_	*r*_x_
q1	0.490	0.771	0.385	q11	0.500	0.833	0.417
q2	0.458	0.833	0.415	q12	0.438	0.792	0.393
q3	0.563	0.792	0.393	q13	0.458	0.875	0.436
q4	0.479	0.792	0.395	q14	0.469	0.729	0.364
q5	0.490	0.688	0.344	q15	0.490	0.813	0.406
q6	0.458	0.792	0.394	q16	0.448	0.729	0.363
q7	0.500	0.792	0.396	q17	0.490	0.854	0.427
q8	0.469	0.771	0.385	q18	0.490	0.771	0.385
q9	0.448	0.771	0.383	q19	0.531	0.813	0.405
q10	0.542	0.833	0.415	q20	0.417	0.667	0.329

KR20 = 0.97; $\bar{p}$ = 0.48.

When the item analyses of the Academic Achievement Test were examined, it was observed that all items in the test form had item difficulty (*p*_j_) indices ranging between 0.42 and 0.56, item discrimination (*r*_jx_}) indices between 0.67 and 0.88, and item reliability (*r*_x_) indices between 0.33 and 0.44, all of which are at a good level. In addition, the KR-20 coefficient (0.97) and the average test difficulty ( $\bar{p}$ =0.48) also indicated good values.

Two example items from the Academic Achievement Test used in the study are provided below:

1. Which of the following is not a correct statement regarding informal education?
  - It is purposeful *
  - It is not carried out within a plan
  - It occurs spontaneously
  - It can take place in any environment and at any time
  - An individual may develop positive or negative behaviors
2. After gaining knowledge in the Social Studies course, a student who decided to become more sensitive to the environment began throwing waste into the trash bin and making an effort to use water more economically.

*Which teaching principle can be said to have been particularly emphasized by the teacher in this lesson?*

- From the known to the unknown
- Relevance to real life*
- From simple to complex
- Topicality
- Clarity.

#### 2.4.2 Learning Motivation Scale in Higher Education

Developed by Erduran Tekin (2023), the Learning Motivation Scale in Higher Education consists of 23 items with three response options (Disagree, Neutral, Agree). The factor loadings of the items are 0.35 and above, and the total variance explained is 50.1%. Furthermore, the Cronbach’s alpha internal consistency coefficient of the scale is reported to be above 0.79.

Only usage permission—not reproduction rights—was obtained for this scale; therefore, illustrative sample items rather than original items are presented.

–*“I prefer not to participate in classroom discussions when I feel uncomfortable.”*

–*“Performing well compared to my peers is an important source of motivation for me.”*

#### 2.4.3 Cognitive Load Scale

The Cognitive Load Scale was developed by Paas and Van Merriënboer (1993) as a rating scale. The scale consists of a single item with a 9-point rating system ranging from very, very low to very, very high. The interpretation of scores obtained from the scale is as follows: 1.00–4.49 = not loaded, 4.50–5.50 = in-between, 5.51–9.00 = loaded. The Turkish adaptation of the scale was carried out by Kılıç and Karadeniz (2004) reporting a Cronbach’s alpha coefficient of 0.78 and a Spearman-Brown split-half reliability coefficient of 0.79.

### 2.5 Data analysis

Prior to conducting analyses on the data obtained from the study, the required assumptions were tested. In this context, the normality assumption was first examined (Table 4).

**Table 4:** Normality analysis.

Measure	Group	Shapiro–Wilk	df	*p*	Skewness	Kurtosis
Academic achievement pre-test	Exp. 1: Cornell	0.935	32	0.055	−0.011	−1.072
	Exp. 2: Parallel	0.942	33	0.077	−0.223	−0.787
	Exp. 3: Digital	0.946	33	0.099	0.042	−0.778
	Control: Sentence	0.956	36	0.163	0.056	−0.553
Academic achievement post-test	Exp. 1: Cornell	0.958	32	0.241	−0.346	−0.502
	Exp. 2: Parallel	0.956	33	0.205	−0.107	−0.621
	Exp. 3: Digital	0.963	33	0.307	−0.479	−0.100
	Control: Sentence	0.962	36	0.256	−0.443	−0.176
Academic achievement retention test	Exp. 1: Cornell	0.936	32	0.056	−0.737	−0.064
	Exp. 2: Parallel	0.952	33	0.157	−0.357	−0.575
	Exp. 3: Digital	0.937	33	0.057	−0.557	−0.460
	Control: Sentence	0.954	36	0.137	−0.241	−0.682
Learning motivation pre-test	Exp. 1: Cornell	0.960	32	0.281	−0.307	−0.811
	Exp. 2: Parallel	0.948	33	0.114	0.126	−1.111
	Exp. 3: Digital	0.951	33	0.138	0.048	−1.128
	Control: Sentence	0.953	36	0.129	0.411	−0.532
Learning motivation post-test	Exp. 1: Cornell	0.979	32	0.757	−0.374	−0.344
	Exp. 2: Parallel	0.957	33	0.207	0.184	−0.967
	Exp. 3: Digital	0.955	33	0.183	0.010	−0.984
	Control: Sentence	0.968	36	0.370	0.357	−0.360
Cognitive load	Exp. 1: Cornell	0.883	32	0.002*	−1.311	2.334
	Exp. 2: Parallel	0.946	33	0.103	−0.549	0.170
	Exp. 3: Digital	0.970	33	0.491	−0.317	−0.358
	Control: Sentence	0.902	36	0.004^*^	−0.986	1.995

* *p* < 0.05.

When the values of the normality analyses presented in the table were examined, it was found that for the variables of academic achievement pre-test, academic achievement post-test, academic achievement retention test, motivation pre-test, and motivation post-test, the normality assumptions were met across experimental and control groups (*p* > 0.05). Additionally, skewness and kurtosis values were within acceptable ranges. However, for the cognitive load variable, normality was not met in the Cornell and Sentence method groups (*p* < 0.05).

For the analysis of the first sub-problem, namely identifying the differences in post-test and retention test scores of academic achievement according to note-taking methods, a mixed ANCOVA was employed (since the main assumptions such as normality, linearity, and homogeneity were met). For the second sub-problem, determining the differences between motivation pre-test and post-test scores, a paired samples *t*-test was conducted (as there were no issues regarding normality). For the third sub-problem, to determine group differences in cognitive load, the Kruskal-Wallis *H* test was used, and for pairwise comparisons, the Mann–Whitney *U* test was applied (as normality was not satisfied). For the fourth sub-problem, the required assumptions for hierarchical regression (linearity, normality, homoscedasticity, VIF < 10) were found to be met.

In the study, it was also tested whether the experimental and control groups were equivalent in terms of pre-test scores of academic achievement and learning motivation, and the findings are presented (Table 5).

**Table 5:** Pre-test scores of academic achievement and learning motivation.

Source		Sum of squares	df	Mean square	*F*	*p*
Academic achievement pre-test	Between groups	1.979	3	0.660	0.211	0.889
	Within groups	406.327	130	3.126		
	Total	408.306	133			
Learning motivation pre-test	Between groups	155.604	3	51.868	1.003	0.394
	Within groups	6725.030	130	51.731		
	Total	6880.634	133			

As shown in the table, the pre-test scores of academic achievements [*F*_(3, 130)_ = 0.211; *p* > 0.05] and learning motivation [*F*_(3, 130)_ = 1.003; *p* > 0.05] across the three experimental groups and one control group were examined, and no statistically significant differences were found. This result indicates that before the experimental process, the groups were equivalent in terms of pre-test scores.

## 3 Results

Within the scope of the first sub-problem of the study, four different note-taking methods (Cornell, Parallel, Digital, and the Sentence Methods) were compared in terms of students’ post-test (T_2_) and retention test (T_3_) academic achievement scores, using the mean (M) and standard error (SE) values, while controlling for pre-tests. In addition, the difference between T_2_−T_3_ and the statistical significance of this difference are presented (Table 6).

**Table 6:** Differences between post-test (T_2_) and retention (T_3_) scores by group.

Group	Posttest *M* (SE)	Retention *M* (SE)	Mean difference (T_2_–T_3_)	Significance
Experimental 1: Cornell NtM	15.9 (2.45)	15.0 (2.78)	−0.9	*p* > 0.05
Experimental 2: Parallel NtM	15.6 (2.10)	14.7 (2.79)	−0.9	*p* < 0.05*
Experimental 3: Digital NtM	13.9 (3.26)	12.7 (3.93)	−1.2	*p* < 0.05*
Control: Sentence NtM	14.0 (2.78)	12.4 (3.09)	−1.6	*p* < 0.01**
Overall mean	14.9 (2.80)	13.7 (3.35)	−1.2	*p* < 0.001***

Adjusted means were calculated with pretest achievement scores as a covariate.

NtM, note-taking method; SE, standard error; *p < 0.05; **p < 0.01; ***p < 0.001.

When academic achievement scores were examined from post-test to retention, a decline over time was observed across the sample. Descriptively, the Cornell group decreased from 15.9 at post-test to 15.0 at retention (*Δ* = −0.9), the Parallel group decreased from 15.6 to 14.7 (Δ = −0.9), the Digital group decreased from 13.9 to 12.7 (Δ = −1.2), and the Sentence group decreased from 14.0 to 12.4 (Δ = −1.6). The overall mean across all groups declined from 14.9 at post-test to 13.7 at retention (Δ = −1.2), and this overall decrease was statistically significant (*p* < 0.001), indicating a general reduction in performance over time. Although some within-group pre–post changes were statistically significant, the time × group interaction was not significant; therefore, these within-group significance tests should not be interpreted as evidence that any note-taking method produced greater forgetting or better maintenance of learning over time. In other words, the data do not support differential retention trajectories across methods; interpretations should be restricted to observed differences at each measurement point.

To formally examine whether post-test and retention scores differed across note-taking methods over time, a mixed-design ANCOVA was conducted, with time (post-test vs. retention) as the within-subjects factor, note-taking method as the between-subjects factor, and motivation included as a covariate (Table 7).

**Table 7:** Mixed ANCOVA results for post-test and retention scores.

Effect	*F*	df	*p*	*η* ^2^ *_p_*
Time	38.98	1,129	<0.001**	0.232
Time × pre-test	1.26	1,129	0.263	0.010
Time × group	1.82	3,129	0.146	0.041
Group	5.62	3,129	0.001*	0.116

*η*^2^*_p_*, partial eta squared; ^*^p < 0.01; ^**^p < 0.001.

According to the mixed-design ANCOVA, the main effect of time was significant [*F*_(1, 129)_ = 38.98, *p* < 0.001, *η*^2^*_p_* = 0.232], indicating that scores decreased from the post-test to the retention test. The time × pre-test interaction was not significant [F_(1, 129)_ = 1.26, *p* = 0.263, *η*^2^*_p_* = 0.010], suggesting that initial achievement did not moderate the change in scores over time. Importantly, the time × group interaction was also not significant [*F*_(3, 129)_ = 1.82, *p* = 0.146, *η*^2^*_p_* = 0.041], indicating that the pattern of change from post-test to retention was comparable across note-taking methods; thus, the results do not support differential forgetting or retention trajectories between groups. The main effect of group was significant [F_(3, 129)_ = 5.62, *p* = 0.001, *η*^2^*_p_* = 0.116], reflecting overall differences in achievement levels across conditions. However, consistent with the pairwise comparisons, between-group differences at the retention test were selective: only the Cornell versus Sentence contrast was statistically significant, whereas the remaining pairwise comparisons were not. Overall, note-taking method was associated with differences in achievement level, but the time-related decline in scores was observed across all groups.

To further examine group differences at each measurement point, Bonferroni-corrected pairwise post-hoc comparisons of the adjusted means were conducted for the post-test (T_2_) and retention (T_3_) scores. For the post-test, none of the six pairwise contrasts between note-taking methods reached statistical significance after Bonferroni correction (all p_Bonferroni ≥ 0.05), although the Cornell (M_adj = 15.9, SE = 2.45) and Parallel (M_adj = 15.6, SE = 2.10) groups showed descriptively higher adjusted means than the Digital (M_adj = 13.9, SE = 3.26) and Sentence (M_adj = 14.0, SE = 2.78) groups. For the retention test, the Cornell method (M_adj = 15.0, SE = 2.78) yielded significantly higher scores than the Sentence method (M_adj = 12.4, SE = 3.09), whereas no other pairwise comparison remained significant after Bonferroni adjustment (all p_Bonferroni ≥ 0.05). Taken together, these findings indicate that between-group differences were limited: no pairwise contrasts were statistically significant at the post-test after Bonferroni correction, and at the retention test only the Cornell versus Sentence contrast remained significant. Accordingly, the only statistically supported method-related difference at the retention test was the Cornell versus Sentence contrast.

Within the scope of the second sub-problem of the study, the learning motivation of the students was measured according to the note-taking method they used, and a paired-samples *t*-test was conducted to analyze the difference between pre-test and post-test motivation scores for each method (Table 8).

**Table 8:** Results of the paired-samples *t*-test regarding the pre-test and post-test motivation differences of students according to note-taking methods.

Methods	Measurement	$\bar{X}$	*N*	sd	*t*	df	*p*
Experimental 1: Cornell NtM	Pre-test	44.25	32	5.80	−3.57	31	0.001*
	Post-test	46.31	32	6.85			
Experimental 2: Parallel NtM	Pre-test	45.76	33	7.32	−4.41	32	<0.001**
	Post-test	47.52	33	7.05			
Experimental 3: Digital NtM	Pre-test	43.30	33	8.64	−0.99	32	0.329
	Post-test	43.85	33	8.50			
Control: Sentence NtM	Pre-test	43.00	36	6.71	0.42	35	0.675
	Post-test	42.86	36	7.20			

^*^p < 0.01; ^**^p < 0.001.

According to the results of the paired-samples t-test, students in the experimental groups who used the Cornell method [*t*_(31)_ = −3.57, *p* = 0.001] and the Parallel method [*t*_(32)_ = −4.41, *p* < 0.001] showed a statistically significant difference between their pre-test and post-test motivation scores. This finding indicates that both methods significantly increased students’ learning motivation. In contrast, for the Digital note-taking method [*t*_(32)_ = −0.99, *p* = 0.33], which was one of the experimental groups, and for the sentence method [*t*_(35)_ = 0.42, *p* = 0.68] which was the control group, no significant difference was found between pre-test and post-test motivation scores. Therefore, it can be concluded that the Cornell and Parallel methods were effective in enhancing students’ learning motivation, whereas the Digital and Sentence methods did not demonstrate a significant effect.

Within the scope of the third sub-problem of the study, the cognitive load levels during the note-taking process were measured according to the note-taking methods used by the students, and a Kruskal-Wallis *H* test was conducted to analyze the differences between the methods (Table 9).

**Table 9:** Results of the Kruskal–Wallis *H* test on the differences in cognitive load levels according to note-taking methods.

Group	*N*	Mean rank	*H*	df	*p*	Mann–Whitney *U* Comparisons
(1) Experimental 1: Cornell NtM	32	64.31	9.671	3	0.022^*^	2 > 3 4 > 3
(2) Experimental 2: Parallel NtM	33	78.52				
(3) Experimental 3: Digital NtM	33	51.82				
(4) Control: Sentence NtM	36	74.61				
Total	134					

* *p* < 0.05.

According to the Kruskal-Wallis *H* test results for the cognitive load variable by note-taking method, a significant difference was found among the groups [*H*_(3)_ = 9.671, *p* = 0.022]. Examination of the mean rank values revealed that the lowest cognitive load was observed in the Digital method (mean rank = 51.82), while the highest values were found in the Parallel method (mean rank = 78.52) and in the control group using the Sentence method (mean rank = 74.61). The mean rank of the Cornell method was determined as 64.31. Mann–Whitney *U* tests conducted to determine the source of the differences indicated that the Digital method experienced significantly lower cognitive load compared to both the Parallel method (2 > 3) and the control group (4 > 3).

Within the scope of the fourth and final sub-problem of the study, the results of the hierarchical regression analysis regarding the predictive power of motivation and cognitive load variables on retention levels according to note-taking methods are presented (Table 10).

**Table 10:** Hierarchical regression analysis results by note-taking method.

Method	*R*	*R2*	*F*	Learning motivation		Cognitive load	
				*β*	*p*	*β*	*p*
Experimental 1: Cornell NtM	0.50	0.25	10.12	0.50	0.003*	0.12	0.459
Experimental 2: Parallel NtM	0.60	0.36	17.66	0.60	<0.001**	0.00	0.989
Experimental 3: Digital NtM	0.54	0.28	12.01	0.54	0.001*	0.24	0.118
Control: Sentence NtM	0.51	0.26	12.07	0.51	0.001*	−0.006	0.969

*p < 0.01; **p < 0.001.

According to the hierarchical regression analyses, motivation was found to be a strong and significant predictor of retention across all note-taking methods. In the Cornell, Digital, and Sentence methods, motivation had a similar level of effect on retention, whereas in the Parallel method this effect was stronger (*β* = 0.60, *p* < 0.001). This pattern suggests that the relationship between motivation and retention may be more pronounced for students using the Parallel method; however, these results should not be interpreted as evidence that the Parallel method increases motivation or retention relative to other note-taking methods. In contrast, cognitive load was not significantly associated with retention in any of the note-taking methods, showing only a positive but non-significant tendency in the Digital method. Overall, the findings indicate that motivation—rather than cognitive load—shows the most consistent association with retention in the present study.

## 4 Discussion

In this study, performance differences between note-taking methods were limited, and interpretations must be aligned with the statistical pattern of results. At the retention measurement, only the Cornell method yielded a statistically significant advantage over the basic Sentence method, whereas no other pairwise comparisons remained significant after correction. Although the Cornell and Parallel/two-column groups showed descriptively higher post-test and retention means than the Digital and Sentence groups, the only statistically supported difference in retention test performance was confined to the Cornell–Sentence contrast. From a theoretical perspective, features of the Cornell format—such as segmenting notes, separating cues from explanations, and requiring summarization—may encourage generative processing and meaningful organization, potentially reducing surface-level transcription and supporting conceptual understanding. By contrast, although students using digital note-taking reported lower cognitive load, this did not correspond to statistically supported benefits in achievement or retention test performance, suggesting that perceived effort alone may not be sufficient to yield measurable learning advantages in this context.

The observed Cornell–Sentence difference at the retention test performance is consistent with empirical work suggesting that guided note formats can support performance. Particularly, findings indicating that guided notes can improve exam and quiz performance (Krapf and Pfefferkorn, 2022) support the organizational and processing benefits offered by frameworks such as Cornell or two-column notes. For instance, study conducted at the higher education level showed that the use of guided notes positively influenced academic performance by promoting attention to key content and fostering active engagement (Krapf and Pfefferkorn, 2022). Similarly, a quasi-experimental study involving nursing students reported that the Cornell note-taking method significantly enhanced both the quality of notes and learning performance (Amhout et al., 2023).

The study’s findings regarding the note-taking medium—digital vs. non-digital (handwritten)—are also supported by the literature. A recent meta-analysis by Flanigan et al. (2024), which reviewed 24 studies, found that although more text can be produced via keyboard, handwritten and subsequently reviewed notes demonstrated a small-to-moderate advantage in overall academic performance. However, another meta-analysis by Voyer et al. (2022) suggested that when distractors and task conditions were controlled, the performance difference between digital and non-digital note-taking diminished or disappeared. These findings imply that the primary determinant of learning outcomes is not the medium itself but rather the structural quality of the notes (e.g., Cornell, guided) and the cognitive operations employed. Indeed, while Mueller and Oppenheimer (2014) initially suggested that keyboard use increases the tendency for verbatim note-taking, which may be disadvantageous for conceptual tasks, their subsequent replication studies revealed inconsistent results (Morehead et al., 2019; Mueller and Oppenheimer, 2014).

From the perspective of Cognitive Load Theory (CLT), the pattern observed for digital note-taking—lower perceived cognitive load without statistically supported advantages in achievement or retention test performance—can be interpreted in a theoretically plausible way. CLT distinguishes intrinsic, extraneous, and germane cognitive load (Sweller, 1988; Sweller et al., 1998) and emphasizes that reductions in perceived load do not necessarily improve learning if they coincide with reduced germane processing (Sweller et al., 2011). Digital note-taking can facilitate rapid, verbatim recording, which may feel easier and reduce perceived demands, but may also limit generative processing and elaboration. Consequently, learners may report lower load while engaging in fewer cognitive operations that typically support deeper understanding. This interpretation is consistent with evidence that subjective cognitive-load ratings do not always map directly onto performance outcomes (Krieglstein et al., 2022) and that structured or collaborative note-taking may alter different load components depending on instructional design (Costley and Fanguy, 2021). Overall, the present findings suggest that lower perceived cognitive load, by itself, is not sufficient to guarantee higher post-test or retention test performance.

With respect to motivation, the analyses indicated significant pre–post increases in motivation for the Cornell and Parallel note-taking methods, whereas no significant pre–post change was observed for the Digital or Sentence methods. Prior research similarly highlights that motivational factors are meaningfully related to retention test performance (MacIntosh and Asghar, 2025). From a self-determination perspective, structured formats such as Cornell or two-column notes may support engagement by providing clearer task structure and opportunities for autonomy in organizing information; however, this interpretation should be tested directly in future work.

This interpretation can also be discussed in light of the Desirable Difficulties framework (Bjork and Bjork, 2011), which proposes that certain forms of cognitive effort—although experienced as more demanding—can support deeper encoding and improve later performance. In the present study, structured note-taking formats (particularly Cornell) may have encouraged more generative and elaborative processing, which could help explain the selective advantage observed at the retention test performance. However, because the time × group interaction was not significant, these findings should not be interpreted as evidence that any method produced different rates of forgetting or systematically “maintained learning better” over time. Likewise, although the digital method was associated with lower perceived cognitive load, the results do not provide statistically supported evidence that digital note-taking yielded inferior retention relative to other methods; rather, any method-related differences at the retention test were limited to the Cornell versus Sentence contrast.

Finally, it may be posited that combining note-taking methods with appropriate structural support and learning conditions can influence the extent to which students benefit from note-taking. Prior research highlights several moderators—such as control of distractions, opportunities for note review, assessment format, and explicit instruction in schematic note-taking—that shape learning outcomes. In this context, studies have reported that implementing Cornell or two-column structures within digital environments can mitigate potential drawbacks sometimes observed in keyboard-based note-taking (Luo et al., 2018; Shell et al., 2021). Consistent with this possibility, the present findings show that the digital method was associated with lower perceived cognitive load but did not yield statistically supported advantages in post-test or retention test performance. One plausible explanation is that, in the absence of structural scaffolding, learners may be more likely to engage in verbatim transcription (Morehead et al., 2019); however, this mechanism was not measured directly and should be tested in future work.

## 5 Limitations and future studies

This study has several limitations that should be acknowledged. First, the sample consisted of 134 s-year pre-service teachers selected through purposive homogeneous sampling, and although participants were randomly assigned to groups, the sample itself was not randomly drawn, which limits the generalizability of the findings. The study did not consider demographic differences such as age, as this was beyond the scope of the research design. Moreover, although the sample size was sufficient for detecting medium-to-large effects, no *a priori* power analysis was conducted; therefore, the absence of a formal power calculation constitutes a methodological limitation, particularly for detecting small effects. The experimental intervention lasted only 5 weeks, and the retention measure was administered once, 4 weeks after the post-test, preventing conclusions about longer-term or shorter-term retention patterns. In terms of cognitive load, only the average perceived load at the end of the intervention was collected, and variations across sessions were not measured. Another limitation is that the study did not include a mediation analysis to examine whether motivation acted as an intermediary variable between note-taking method and learning outcomes, despite motivation increasing significantly in the Cornell and Parallel groups. In addition, no systematic analysis of note quality (e.g., completeness, elaboration, correctness) was conducted, and therefore the potential mediating role of note quality could not be examined; future studies should incorporate validated note-quality coding schemes to explore these relationships. Finally, because standardized note-quality scores were not collected, group-specific correlations between constructs could not be calculated.

Drawing on these limitations, several directions for future research and instructional practice can be proposed. Future studies should employ designs that disentangle the effects of note-taking method (e.g., Cornell, parallel, sentence) from the note-taking medium (paper vs. digital), control the frequency of note review, and include multiple retention intervals. Mixed-methods studies could provide deeper insights into how subjective cognitive load interacts with different task types and how note quality mediates learning outcomes. Instructionally, note-taking training should emphasize not only taking notes but also how the notes are structured—for example, through Cornell or parallel formats—and digital tools should incorporate cues, prompting sections, and summary spaces to address structural limitations. Course designs that encourage students to personalize, revise, and elaborate their notes, along with practices that support autonomy, competence, and relatedness, may further enhance motivation and lasting learning.

## 6 Conclusion

This study compared note-taking methods on post-test and four-week retention performance while controlling for prior achievement. Although an overall method effect was observed, the non-significant time × group interaction indicates that changes over time did not differ across methods, precluding claims of differential forgetting. Post-hoc tests showed no significant pairwise differences at post-test; at retention, only Cornell outperformed Sentence. At retention, the only significant pairwise difference was Cornell versus Sentence.

Motivation increased significantly only for the Cornell and Parallel methods, whereas no significant change was observed for the Digital or Sentence methods. Despite lower perceived cognitive load in the Digital method, no statistically supported performance advantage emerged. Overall, motivation showed the most consistent relationship with retention scores, underscoring its relevance for retention test performance.

Overall, three main conclusions emerge from the present study:

1. The findings suggest that the structure of note-taking may be more consequential for learning outcomes than the medium itself. In particular, the only statistically supported difference in retention test performance was observed for the Cornell versus Sentence comparison, indicating that structured formats can be beneficial across different contexts, including digital environments, when appropriately implemented (Flanigan et al., 2024; Voyer et al., 2022).
2. Motivation showed a consistent association with retention scores across note-taking methods, underscoring its importance for retention test performance rather than implying a direct causal or predictive role (Cerasoli et al., 2014; Richardson et al., 2012).
3. With respect to cognitive load, the results align with theoretical accounts suggesting that learning may benefit when unnecessary (extraneous) load is reduced while meaning-oriented processing is maintained; however, cognitive load alone did not emerge as a significant determinant of retention in the present data (Krieglstein et al., 2022).

Pedagogically, the findings support the value of explicitly teaching structured note-taking routines in the classroom rather than leaving note-taking to students’ spontaneous practices. Formats such as Cornell and Parallel/two-column notes provide teachable steps (e.g., identifying cues, organizing content, and summarizing) that teachers can model, scaffold, and reinforce across subjects. Professional development may therefore benefit from emphasizing not only the allowance of note-taking but also explicit instruction, including the use of templates and formative feedback to help students develop more effective strategies.

At the same time, the current sample and intervention duration limit generalizability. Future research should examine these methods across different subjects, longer durations, and alternative digital implementations, ideally using longitudinal designs to test whether early training produces sustained benefits in motivation and achievement. Within these bounds, the present results suggest that method-related differences at the retention test were limited (i.e., the Cornell versus Sentence contrast), and that structured instruction in note-taking may be a promising, testable approach for supporting students’ learning outcomes.

## Data Availability

The raw data will be made available by the author(s) upon reasonable request, without undue reservation.
